# How is the New Public Management applied in the occupational health care system? - decision-makers' and OH personnel's views in Finland

**DOI:** 10.1186/1478-4505-9-34

**Published:** 2011-08-31

**Authors:** Hanna Hakulinen, Sari Rissanen, Johanna Lammintakanen

**Affiliations:** 1 Health and Work Ability, Finnish Institute of Occupational Health, P.O. Box 93 FI-70701, Kuopio, Finland; 2 Department of Health and Social Management, University of Eastern Finland, P.O. Box 1627 FI-70211 Kuopio, Finland

## Abstract

**Background:**

In many countries occupational health care system is in change. Occupational health studies are mainly focused on occupational health substance and content. This study offers new perspectives on municipal OHS and its operations from management perspective.

**Aim:**

The aim of this study is to analyse how New Public Management (NPM) doctrines are applied in the Finnish occupational health care system (OHS). The main focus is to describe and compare the views of decision-makers' and OH workers within the framework of NPM.

**Methods:**

The data were collected by semi-structured interviews from 17 municipal decision-makers' and 26 municipal OH workers. Data was analyzed by examining coded data in a theory-driven way according to Hood's doctrine of NPM.

**Results:**

The doctrines were not as compatible with the OH personnel view as with the decision-makers' view. Decision-makers and OH personnel highlighted the strict criteria required for operation evaluation. Moreover, decision-makers strongly accentuated professional management in the public sector and the reorganization of public sector units. These were not equally relevant in OH personnel views. In OH personnel views, other doctrines (more attention to performance and accomplishments, emphasizing and augmentation of the competition and better control of public expense and means test) were not similarly in evidence, only weak evidence was observed when their importance viewed as medium by decision-makers. Neither of the respondents group kept the doctrine of management models of the private sector relevant.

**Conclusions:**

The NPM and Hoods doctrine fitted well with OH research. The doctrine brought out view differences and similarities between decision-makers and OH personnel. For example, policymakers highlighted more strongly the structural change by emphasizing professional management compared to OH personnel. The need for reorganization of municipal OH, regardless of different operational preconditions, was obvious for both decision-makers and OH personnel. The adaptation of more clarify management to a municipal context is not trouble-free. The municipality systemic structure, complex operational environment, and reconciliation of political and officer authority set challenges to management of municipalities.

## Background

The aim of this study is to analyse how New Public Management (NPM) doctrines are applied in the Finnish occupational health care system (OHS). The main focus is to describe and compare the views of municipal decision makers and OH workers within the framework of NPM.

The arguments for this study are the following. New public management ideas were adapted in Finnish public administration quite late during the 1990s, introducing decentralisation, simplification of the central administration, stricter control of expenses, public utilities, outsourcing of the services, and renewals to improve the client-oriented operation. These trends still have a strong influence in the Finnish occupation health system. In addition, the content of Finnish occupational health has needed reconsideration based on changes in the working life. For instance, a knowledge-intensive working life with irregular working contracts and increasing skill demands requires a new orientation and new working methods in occupational health. In addition to content development, the structures of OHS are being changed. The division of labour between private and public service providers is changing, the number of private actors has increased, and this has challenged the traditional ways of working for public services providers.

Previous OH studies are focused mainly on OH substance and content (e.g. medical treatment, physical examinations) [e.g [1-3]]. We have only very low evidence of management studies of OHS [e.g [4,5]]. This study offers new perspectives on municipal OHS and its services, which are at a turning point. In addition, this study can be interpreted as an attempt to test and develop New Public Management ideology in the context of occupational health. The main focus in the NPM reform wave is that a more market orientation in the public sector will lead to greater cost efficiency for governments, without having negative side effects on other objectives and considerations. Although there are several alternative public administration theories, in this study the use of NPM is supported by its connections to the current aim of seeking change and modernisation in a Finnish occupational health care context.

### The cornerstones of New Public Management (NPM)

The scholarly debate about NPM reforms has increasingly been influenced by what Premfors [6] labels the "structured pluralism story", focusing on variation to the extent to which different countries have implemented NPM reforms [7-14]. Most of the literature on NPM reforms analyse them as public administration reforms. However, when it comes to reforms such as the introduction of quasi-markets in the health care sector or private providers of elderly care, NPM reforms become part of the political battle over the welfare state. This need to analyse NPM reforms within a welfare-state context is particularly important when dealing with the Scandinavian countries. The literature on different welfare-state regimes has documented that countries differ considerably in the extent to which they offer their citizens health and welfare services such as elderly care and childcare and also very much in the way such services are provided [15,16]. In Scandinavia, such services are extensive and mainly publicly funded and provided, whereas in continental European countries, such services are less extensive, publicly funded, but generally not publicly provided. The implication of these differences for the NPM debate is clear. The public sector, which should be reformed along NPM lines, is different from country to country. In Scandinavia, the public sector includes the extensive service welfare state, and therefore NPM reforms must be analysed within the broader welfare-state context [17].

Despite early efforts to place the debates on the shift to New Public Management within broader processes of economic and political restructuring - processes that suggest that various trajectories are possible - much of the literature has tended to assume that the shift is inevitable [18,19]. The characteristics of New Public Management are contested and there is still, in fact, considerable confusion over what New Public Management actually is [19-21].

So the literature on NPM is now quite wide and includes official exhortations to adopt various elements of NPM, academic considerations of the advantages and disadvantages of NPM, and various texts dealing with specific elements of NPM techniques [22]. Within the NPM framework, cross-country examination is also done, but there is still a need for additional country-specific and system-level studies in this field [23].

As earlier stated, binding the NPM discussion to a single perspective is challenging. Table 1 summarises the essential content of NPM as interpreted by various researchers. Some of the researchers interpret NPM widely, but they emphasise different aspects of the concept. First we deal with narrow definitions, and then we focus on the larger definitions.

**Table 1:** Summary of the NPM's essential content.

The content of NPM	Researchers
Three E's as NPM's values: economy, efficiency, effectiveness	Vakkuri & Meklin [24], Pollit [25], Pollit [26], van Wart & Berman [27]

Accountability of action in public services	Boyne [28], Sanderson [29], Lane [30], Aucoin [31], Keating [32], Hartley [33]

Professional management and cooperation based on contracts between different actors. Private sector as a management model. Governance in public sector.	Pollit [25], Moore & Henegan [34] DeLeon [35], Hogget [18], Hood [36], Walsh [37], Lane [38], Pollit [39], Salminen [40], Vedung [41], Kolthoff et al [42], Aucoin [31], Weikart [43], Moon & deLeon [44]

Separation of political and administrative decision-making	Aucoin [31], Hood [35], Walsh [37]

Entrepreneurship of public sector	Box [45], Morris & Jones [46]

Focus on the citizen role	Exworthy & Halford [47], Ahonen & Salminen [48], Piotrowski & Rosenbloom [49], Ezzamel et al [50]

Wide definition of New Public Management covering most of the previous elements	Hood [35], Foster & Plowden [51], Ferlie [20], Hays & Kearney [52], Walsh [36], Hood [53], Hogget [18], Hambleton [19], Pollit [25], Stark [21]

Besides NPM's comprehensive definitions, in the literature NPM is also approached by emphasising the more compact content part of NPM. For instance, van Wart & Bergman [27], Pollit [25], and Vakkuri & Meklin [24] emphasise economy, efficiency, and effectiveness as values of NPM. In that case, the recovery of administration capacity and responsibility of management finances to citizens are considered relevant. Exworthy & Halford [47], Piotrowski & Rosenbloom [49], Ezzamel et al [50], and Ahonen & Salminen [48] emphasise citizen roles in the context of NPM. The citizen concept has enlarged both citizens who make choices and financiers who pay taxes. Pollit [25], Moore & Henegan [34], and DeLeon [35] consider the private sector as a role model for public management and are thus looking for generalisation for the management.

According to Lane [34] on government thinking, NPM is based on two assumptions. First, demand must be separated from supply. The second principle, contestability, is that there must be competition in supply. The conclusion of those two principles is contractualism. Lane says [34] that NPM is the same as the massive use of contractualism in government.

However, Boyne [28], Sanderson [29], Hartley [33], and Keating [32] emphasise accountability as a crucial principle in NPM. Moreover, they state that profit responsibility is very important for public services. Sanderson [29] also emphasises the importance of the increase in supervision. Behind the accountability lies the ambition to improve public action plausibility among citizens [30,28].

On the contrary, several researchers [18,38,39,41,42,44] emphasise more professional management, managerialism, and contractualism between different actors. More professional management and contractualism is expected to be the conclusive factor to maximise the effectiveness of the public sector [38,41]. Moreover, according to Lane [38] and Kolthoff, Huberts and van Den Heuvel [42], the public sector will get more effective administration by exploiting professional management. However, Walsh [38], Hood [53], and Aucoin's [31] central feature of the NPM is the separation of politics and management. Mascarenhas [54] also highlights the ambiguous conception of public servants commensurate with political decision-making.

Public sector entrepreneurship is also an essential part of NPM content [45,46]. Morris & Jones [46] define public sector entrepreneurship as a combination of public and private resources in order to produce additional value for citizens. Theoretically, public sector entrepreneurship stands for the derivation of novel managerialism, seeing that the essential basis of managerialism is related not only to management emphasis but also to the application of private sector management principles and practice to the public sector.

One way to analyse the NPM concept is to make a list of the principles of NPM. For instance, Kolthoff et al [42] introduce in their article two principles of NPM which are distinctly based on Pollit [55] and Walsh [37]. The first principle (the 'Taylorist principle') is based on the adoption of industrial production engineering techniques within the public sector. It is not a rejection of bureaucracy but rather its fulfilment. The second principle is based on the primacy of market-based coordination.

Furthermore, Hays and Kearney [52] emphasise downsizing, managerialism, decentralisation, de-bureaucratisation, and privatisation. For one, Kettl [56] summarises that the reforms were built under six components: productivity, marketisation, service orientation, decentralisation, policy, and accountability.

Hood [14,35,53] has also classified NPM by covering many different aspects of previously mentioned definitions. In Hood's publication can be found the underlying doctrines of NPM: (1) management of public organisations requires professional management skills; (2) strict criteria are required for operation evaluation; (3) more attention to performance and accomplishments; (4) reorganisation of public sector units; (5) emphasising and augmenting the competition in the public sector; (6) management models in the private sector; and (7) better control of public expense and means test.

In summary, since the 1990s the definition and content of NPM are considered to be broader. The conversation on the role of NPM as a global phenomenon has especially increased. Thus, the definition of NPM is considered to vary between the countries and the public administrative sector. Nevertheless, Hood's doctrines provide a good basis for the investigation and determination of this multiform public management phenomenon. As seen in Table 1, Hood's doctrines cover the entire NPM entirety. Therefore in this study, Hood's doctrines [35,53], as enforced, are used to examine NPM in the context of OHS.

### Study context: Description of the OH service system in Finland

In Finland, all employers are obliged to organise occupational health services for their employees according to the Occupational Health Care Act (2002) to ensure that the preventative contents of the services take into account the needs of the working life and personal characters. Occupational health care can include medical care and other health care services. Occupational health services are planned as a cooperative effort at the workplace by occupational health professionals. Occupational health services can be organised in several ways: in municipal health care centres, in private medical clinics, services may be integrated into the enterprise, or enterprises can collaborate in organising the concerted occupational health services. The occupational health services, which are free of charge for employees, are substituted by the employers by The Social Insurance Institution of Finland. The Social Insurance Institution pays 60% of the costs in compensation to employers that have concluded an agreement with their occupational health service on activities to maintain work ability, follow-up, and early support at the workplace. On the other hand, if the employer has not made this agreement, the compensation for the costs of preventive occupational health care is 50%.

In Finland, there are altogether 663 OH units, which have 1.87 million individual clients in 2007 [57]. This is a considerable number of clients in a country with 5.3 million people. There are 130,829 organisations as clients. The coverage of OH is 87% among employees because small entrepreneurs do not need to have a contract-based OH, but they can use the services offered by the public sector at primary health care centres. The total costs of OH were 596.2 million euros in 2008. Municipal OHs accounted for 121.9 million euros of this sum. The entire Finnish health care costs were 15.5 billion euros in 2007 [58]. A total of 209,439 employees (173,900 in municipal health care and 35,539 in private health care) work in Finnish health care, of which 6,900 work in the Finnish OHS [59-61].

One-third of OH units are organised by the municipalities with about 600,000 clients. 91 of 663 units are the only provider of OH services in its area. The system of municipal OH ensures the availability of OH services in the whole country. Private units are for-profit organisations located in larger cities, whereas municipal OH units are not-for profit organisations. Their focus of services is also different: private units provide more medical services and municipal units more preventive services. For instance, the coverage of medical services is much larger in private units (89%) than in municipal units (60%) [57,61].

Management of Finnish health care can be divided into political and operative management. The municipal OHs are steered through national and municipal policy-making processes. Political management consisted of elected policymakers, who are elected for four-year terms. Elected policy makers control the action of municipal OHs, for example, resources and prices through municipal decision-making processes. At the municipal level, municipal managers implement these political decisions in different organisations. By simplifying, municipal managers are leading and organising municipal OH processes as acting as providers of the services, whereas municipal elected policy makers are acting as purchasers of OH services.

Management in health care organisations has traditionally been hierarchical, and this also concerns the management of occupational health care. The management has also been based strongly on professions, especially the medical profession. On the organisational level, management by results and quality management have been the main orientation in management during the last decades. In macro-level management, the provider-purchaser split and client-orientation in management have been the most often-mentioned principles in management. These same principles are similar also in OH management.

The functionality of municipal OH units varies a lot. High-quality state-owned OH companies do exist, but many smaller OH units with few overall resources have difficulty ensuring sufficient services for their clients. In the small OH units, there may be only a part-time occupational health (OH) nurse performing the services, and the shortage of OH physicians complicates the pursuits of these units [57,61]. These problems are most common in sparsely populated peripheral regions such as northern and eastern Finland. To conclude, during the last years the change in Finnish OH has been substantial. In Finland, OH services are increasingly provided in private OH clinics and in larger public or private units. For instance, during 2004-2007, the number of private OH clinics increased by 11% [57]. Therefore, municipal OH units have to reconsider their operations so that they can respond to these changes. These changes reflect the adoption of NPM principles.

## Methods

The data were collected by semi-structured interviews from 17 municipal decision makers and 26 municipal OH workers from central Finland in January and February 2009. Municipal decision makers consisted of elected policy-makers and permanent municipal managers (municipal managers and health care managers). A semi-structured interview was based on themes selected beforehand, while there was no clear structure or order in the questions during the interview [62,63]. This method of collecting material enables interaction between the interviewer and interviewee, making it possible to use questions to extend the understanding of the participants' conceptions [64,65]. The interviews took an average of 44 minutes each. The municipal OH personnel data consisted of the interviews of OH physicians, the OH nurse, the OH psychologist, and OH physiotherapists. Interviews were transcribed almost verbatim.

The coding was carried out with the help of the ATLAS/ti program using open coding (ATLAS/ti 5.0). In the present study, data analysis included six phases: (1) transcribing the data into written form, (2) getting a general impression of the data, (3) searching the data for relevant and adequate expressions related to the research aim, (4) using the expressions to form appropriate units, (5) comparing the appropriate units for their similarity and diversity, and (6) creating the final categories: main categories and subcategories. Data was analysed by examining coded data in a theory-driven way according to Hood's doctrines of NPM. Thus, it was observed to what extent the development and change of municipal OH corresponds to NPM content and which extra components are included in the development and change of municipal OH. The results are introduced by Hood's doctrines and with extra components. After analysing the content, doctrines were evaluated by importance (how often the doctrine appears) and variation (unity) between the groups of respondents.

Although the data was a case of 12 Finnish OH units, this study represents the situation of OHS in one Finnish province. The collected data were rich and provided the answers sought in this study. This kind of study design corresponds to the study's aim of understanding, not interpreting or generalizing respondents' views. So an added value was to more deeply understand the phenomenon in this special context [66].

## Results

Table 2 summarises the results. Certain Hood's doctrines were more important to decision makers than to OH personnel. Decision makers highlighted more professional management skills and reorganisation of the public sector in an OH context than OH personnel. Instead, both groups emphasised the need of strict criteria for operation evaluation. Neither decision makers nor OH personnel pointed out that performance and accomplishments need more attention. Neither decision makers nor OH personnel considered private sector management models relevant in an OHS context. There were two categories that did not fit Hood's doctrines: (1) shared understanding of challenges which was especially important to decision makers, and (2) common goal for action. It's essential to notice that there were some variation in respondents' views, although the importance of doctrine was high. The next results will be presented in detail.

**Table 2:** Summary of results.

	Decision makers		OH personnel	
**Doctrines**	**Importance**	**Variation**	**Importance**	**Variation**

Strict criteria are required for operation evaluation	High	Medium	High	Medium

Management of the public organizations requires professional management skills	High	Medium	Medium	Medium

Reorganisation of Public Sector units	High	Medium	Medium	Medium

Emphasising and augmentation of the competition in the public sector	Medium	Medium	Low	Low

Better control of public expense and means test	Medium	Low	Low	Low

More attention to performance and accomplishments	Low	Low	-	-

Management models in Private Sector	-	-	-	-

**Categories not fitting Hood's doctrines**				

Shared understanding of the challenges in the municipal context	Medium	Medium	Low	High

Common goal for action	Medium	Medium	Medium	Medium

Importance and variation of doctrine for respondents, scale: Low-Medium-High.

### Hood's Doctrine: Strict criteria are required for operation evaluation

According to decision makers, there was a clear need for development of accountability and evaluation in occupational health care services. Regional collaboration could be one possible way to arrange uniform quality and a service criteria of "Good occupational health practice" for the entire population of working age.

Decision makers thought that OH requirements for levels and the indicator of results should be based on cooperation in OH between municipality and employers. In addition, it is considered important that the indicator of results is based on the needs of municipalities and employers. Cooperation with employers and its development on the whole was a significant challenge from the perspective of both planning and especially OH follow-up and evaluation.

Decision makers expected more transparent objective settings to both OH services and the outcome of services. Based on the opinion of decision makers, the focus of OH operation should be more preventative and workplace-oriented. However, the possibility for companies to receive agreements of medical treatment was also regarded as important. Decision makers expected to get information on the effectiveness of OH to support their decision making and promote the welfare of the employees. This, however, requires developments in follow-up and evaluation of operations.

"I would think that one important target for development would be that we could meet the needs at the working places, as well as possible, the needs of the client firms. After all, that determines the metrics activities." (OH personnel)

"If we talk about goals, they are not always clear. Sometimes I also feel that OH is no longer in the workplace. And then the data and report of our workers would be nice to get." (decision makers)

OH personnel considered especially corporate client cooperation and workplace-oriented services as a development challenge. Corporate client cooperation was considered as a foundation for accountability. Moreover, the challenges of accountability were emphasised in the OH units' internal operations. Productisation, pricing, marketing, and net budgeting were development challenges.

OH personnel saw that quality of operation and quality work produce criteria for service evaluation. Quality work was standardising and strengthening work practices. However, most of the OH personnel thought that the quality of OH needs still improvement. Only a small part of OH personnel thought that the quality of OH is on a good level.

"Sure, there are things to develop in the quality of our activities, especially the level of quality and monitoring. There is a lot of variation and no uniform working practices." (OH personnel)

"The other has a good practices and in cooperation we can certainly improve the quality of health care. Yet the effectiveness of that, yes, it should be assumed from a successful client cooperation." (decision makers)

### Hood's Doctrine: Management of public organisations require professional management skills

According to decision makers, change in municipal OHS is essential. The unclear role and lack of leadership in OH were considered to be the most significant problems. Regional collaboration was conceived as a possibility for network enabling specialisation and cost-effectiveness. However, regional collaboration was regarded as a possible way to get more responsible, coordinated, and goal-oriented management for future services.

"It would be more effective cooperating with others, and one would perceive this role better in the occupational health care system and the municipality." (Decision makers)

According to decision makers, a more specified management system for OH is required. Especially the OHS detachment from other primary health care was considered problematic. Decision makers thought that OH also has an ambiguous role in the municipal environment. The lack of OH unit management, internal development work of OH units, and lack of resources for these were regarded as the most important challenges for OH units. Decision makers also evaluated their own role in OH management. Decision makers thought that they lack the capability for decisions due to their slight OH knowledge and minor visible role of OH in municipal policymaking processes.

"There are problems in cooperation. Yes, change is necessary in order to strengthen customership and management. This gives directors an impartial occupational health service and otherwise supports the specialisation." (Decision makers)

The management of an OH unit was conceived as a challenge, and this challenge was not recognised in all OH units. Allocation of time between management work and everyday OH activities was seen as problematic. Due to the lack of full-time OH managers, the management reserved more work time than was planned. Management decentralisation in different occupational groups was also problematic in almost all OH units. Especially decentralisation of power complicated OH units' own operation planning.

"We do not have a real leader. We do everything together or whoever has the time." (OH personnel)

"One should find time for leadership. Time goes to basic occupational health activities. The workload of management is always surprising." (OH personnel)

"Changes are eagerly anticipated, if only strength could be gained for more professional management. If we would get a real manager, now we are going professional." (OH personnel)

Among OH personnel, the required change in management was considered to lead to more active and systematic management. Moreover, the administrative change was thought to clarify the management. On the other hand, structural change was thought to provide both more authority to OH superiors and more responsible and influential management to OH units.

### Hood's Doctrine: Reorganisation of Public Sector units

The majority of decision makers saw change as a prerequisite for the existence of municipal OHS. According to decision makers, municipal OH requires reorganisation. Reorganisation was considered to include the separation of municipal OH from primary health care in order to create a bigger entity or a public utility. Thus, many advantages were expected: especially security of the OH services in the whole province, cost-effectiveness and its effect on making OH management easier. It was seen that this would also clarify the roles of those people (OH personnel, employers, decision makers, and managers of primary health care) who are cooperating in municipal OH. Decision makers also appreciated the separation of purchaser-provider from each other in order to clarify their roles. *"Yes, it seems that change and reorganisation is necessary. Occupational health services unit will be able to act more resiliently." (decision makers)*

"...and then some; the fact that the removal of basic occupational health care could help us too, the purchaser and the producer could be easily distinguished from each other." (decision makers)

Regional collaboration appeared in the context of networking by both allocation of special know-how, resources, and cost-effective operation. The need for disconnecting OH from primary health care strengthened the above-mentioned cooperation. However, decision makers also approached this doctrine through internal development of OH, productisation, and cost-effectiveness.

OH personnel did not consider reorganisation of public sector units as important as decision makers. The public utility of OH units was regarded as strengthening and improving municipal OH management efficiency. However, for several OH personnel, structural change was regarded as a threat and the most threatening factors were related to the concern for the OH units' future and to changes in occupational health processes.

"Sure, this seems to be that public utility is modernity, and this will surely bring efficiency in our operations. New operating models, which are quite welcome, just seems to be the lifeblood." (OH personnel)

"The future of occupational health care restructuring, and yes, wondering whether it affects the core of this, and there's always such a combination of stronger roles." (OH personnel)

### Hood's Doctrine: Emphasising and augmenting competition in the public sector

The respondents focused on the cooperation between private and municipal OH in this doctrine. Decision makers perceived cooperation between private and municipal OH mainly by way of functional differences and competition. The functional differences were observed in service supplies and basis for focus. Private OH was considered to be focused on medical treatment and municipal preventative occupational health care. It was recognised that private OH is both gaining ground in metropolitan areas and slowly replacing municipal OH. Regional collaboration was seen as a necessity in municipal OH and structural adjustments. Thus, bigger and (in the operation model point of view) more cost-effective OH units could be created. It was considered that this is the only way to compete against private OH.

"Is it then just a private alternative to the roles of going crazy, so be regarded as private. After all, those differences in a particular activity, but municipal (occupational) should wake up the competition." (decision makers)

"It seems that unless the municipality re-organised occupational health care, private power, at least in the growth centres overthrow the municipal health care." (decision makers)

Competition inside of municipal OH was not highlighted among OH personnel. Instead, cooperation with private OH units was considered mainly in terms of competition. Private OHS was seen as a threat to municipal OHS. Prejudices were also affiliated against private OH, for instance, as an employer. In contrast to the decision makers, municipal OH personnel did not count competition as a matter that would produce more positive, good quality and cost-effective OH services. Furthermore, in the future, competition against private OH was a concern to OH personnel, and privatisation of Finnish OHS caused some anxiety.

"It is the private occupational services who are fishing for clients, but the service they provide is quite acceptable. Therefore, it is questionable professional job." (OH personnel)

"Of course it (the private occupational service) is a clear threat to our municipal services. That is co-illustrated by the competition." (OH personnel)

### Hood's Doctrine: Better control of public expense and means test

Decision makers approached this doctrine primarily within the OHS multiple change. Better control of expenses and evaluation of resources' requirements were the key factors that were considered to be attainable by implementing structural changes and merging OH units into larger organisations. The lack of resource evaluation was a consequence of unclear cooperation between OHSs and primary health care services and of inadequate collaboration with the corporate client.

"Of course, also interested in economic issues; seems to be clear that a larger unit would get better control of expenditure and also the size of the resource is more relevant." (decision makers)

The effectiveness of OH units was emphasised by decision makers. Effectiveness was considered from the points of view of the public economy and operations. It was thought that this effectiveness could be attainable by producing cost-effectiveness, net-budgeting, and a means test. From the services' point of view, effectiveness was seen above all by means of the OH basis for focus. Decision makers felt that OH services would be more influential if more resources were channelled to preventative work instead of medical treatment. It was stated that by focusing on OH, small resources could also be used to yield quality OH services.

"If you still come back to effectiveness...after all, since there is a clear link between economic activity and occupational health care, professionals should then consider what is really profitable". (decision makers)

Although the great majority of OH personnel saw the structural change as a necessity for ensuring more quality, cost-effective, and multi-professional OH, it was not highlighted in this doctrine. Instead, they considered reasoning of this kind as a threat.

### Hood's Doctrine: More attention to performance and accomplishments

Neither the decision makers nor OH personnel stated strongly that more attention should be paid to performance and accomplishments.

### Hood's Doctrine: Management models in Private Sector

It was observed that the adaptation of the private sector management models was not essential for municipal OH and its development in both respondents' groups.

### Categories not fitting Hood's doctrines

Two main categories of the NPM in OHS data did not fit the contents of Hood's doctrines. These were: Shared understanding of the challenges in a municipality context and a common goal for action. The respondents didn't share a common understanding of the development challenges. According to decision makers, the changes and challenges of the society came up strongly in the future of OHS. These changes and challenges include, for example, OH's support during in a recession, OH as a support in the change, and challenges of an ageing population. OH personnel did not highlight the change in ambient society as strongly as decision makers. Their talk about the future focused on the OH units' internal matters (for example, resources and multi-professional cooperation) and conceivable structural changes.

The goal differed between the decision makers and the workers. Decision makers spoke about the meaning of OH and not about the influence of OH. They thought that the meaning of OH is to enhance work ability and work well-being. They also felt that OH is an employment benefit for employees. To OH personnel, the most significant influential factor is the functionality of OH personnel, for example cooperation in the workplace and preventative work. The client was generally regarded as an influential indicator of OH.

## Discussion and conclusions

The aim of this article was to analyse how New Public Management (NPM) doctrines are applied in the Finnish occupational health care system (OHS). The main focus was to describe and compare municipal decision-makers' and OH workers' points of view within the framework of NPM, especially exploiting Hood's [14,35] classification. Firstly, the validity and some limitations of this study will be discussed and then conclusions will be summarised.

Interviewees were informed before an interview about its content and the voluntary nature of their participation. Informal consent was obtained from all interviewees beforehand. Anonymity was assured and the individual interviewees cannot be identified. The role of researcher was neutral during the interview and the themes used were congruent in both groups.

However, there are some limitations of this study. The results are context-related to the Finnish occupational health care system and therefore the results have to be interpreted and transferred with care. In addition, the NPM approach was used in this study as a theoretical framework, but this choice can be criticised. Alongside NPM, another trend of ideas that emerged behind the structural and functional changes is the New Governance approach. When observing the current management ideology of the Finnish municipal OHS, the framework provided by NPM to analyse development and change was proven adequate. Still, the operationalisation of NPM on a practice level is somewhat problematic. In this study, Hood's doctrine was used as an attempt in this operationalisation process. Hood's definition provided a good basis to analyse how NPM doctrines are applied to the Finnish occupational health care system. The doctrines revealed the state of Finnish municipal OH, although some new themes emerged to fulfil Hood's framework.

The results can be summarised as follows: Among both decision makers and OH personnel, the doctrine of strict criteria required for operation evaluation came up strongly. Moreover, decision makers strongly accentuated the doctrines of professional management in the public sector and the reorganisation of public sector units. These doctrines were not equally relevant in the view of OH personnel (medium importance). Decision makers' thoughts about OH's municipal development was more similar to doctrines than the view of OH personnel. Neither group of respondents felt that the doctrine of private management models was relevant. In the respondents views, there were some relevant main categories not included in Hood's doctrines. Altogether, the NPM and Hood's framework fit well with OH research. The framework brought out both municipal OH's development and need for change as well as differences in the views of decision makers and OH personnel.

Based on the NPM framework and the results of this study, some relevant issues can be summarised. Firstly, clear authority for the OH management was seen to clarify the challenging role of OHS related to municipal decision makers, other primary health care, and the employers. However, the adaptation of more clarify management to a municipal context is not trouble-free. The system structure of municipalities, complex environment, and reconciliation of political and managerial authority bring challenges for OH management. In this study, a lack of coherent management appeared on different levels by different actors in their strategic thinking. Municipal managers had a cohesive vision of the development of municipal OH, whereas elected policy-makers approached the development and change more pragmatically, and more variation was observed in their views of change. According this study, we can conclude that future strategy was not clear for OH personnel.

Secondly, high professionalism in the personnel generates special demands for the management and development of OH. Administration reform accordant with NPM often highlights the confrontation between professionalism and managerialism. OH personnels' views on development leaned much on professionalism - expertise and education - which are not the issues introduced by decision makers. Instead, decision makers brought out the demands for effectiveness in NPM and features of managerialism. However, professionalism and managerialism/NPM can operate side-by-side. It is essential to find a middle-of-the-road path between self-direction and freedom allowed by professionalism and performance measurement and marketing terms entailed by NPM in the development of municipal OH.

Thirdly, the need for reorganisation of municipal OH, regardless of different preconditions, was obvious for both decision makers and OH personnel. More entrepreneurship approach and a clear split between purchaser and provider could be one solution to municipal OH development, as well as a backlash against competition with private OH. According to our results the change of Finnish municipal OH contains a lot of elements that fit with NPM, for example the growth of OH personnel authority, more well-defined responsibilities, and fewer decision-making steps.

Fourthly, the NPM framework also brought out differences between the OH personnel's and decision-makers' worlds in terms of OH. This is significant information since the starting point of change and development is different among workers as compared to decision makers. It is essential that the following issues are carefully considered: how these two worlds confront each other, how OH personnel adjust to change, and what is needed for municipal OH to develop. In addition, it's important to think about the position of different decision makers in different kinds of welfare systems. In this study, we found different political and professional views on the trend of OH development. It is important to enhance better communication between elected policy-makers, municipal managers, and OH personnel to achieve a common vision of the development of OH. A prerequisite for this cooperation is elected policy-makers' and municipal managers' knowledge of OH needs and possibilities and vice versa.

Fifthly, one aim of this study was to test and develop the New Public Management ideology in the context of OH. For this system-level and country-specific analysis, Hood's doctrine provides a good framework. However, it seems that some new elements could be added to Hood's doctrine and some existing elements were not considered important by the respondents. Especially the common goal for action category could be something which should be reconsidered to fulfil Hood's doctrine or the entire New Public Management ideology.

In comparison to other countries, the NPM reform has been only partly implemented in Finnish OHS. Whereas in some other countries the wave of NPM may be in the past, it is still present in Finnish health care. The NPM ideology has been the driver for public sector change. NPM has challenged some of the old-fashioned models of management as seen in Finnish health care. In countries where political decision makers have a role in health care, their role should be considered when implementing management models. In the global context, different countries are in different phases in developing their OHS and NPM. Those countries that are developing their OHS would benefit from research-based information of the NPM.

## Competing interests

The authors declare that they have no competing interests.

## Authors' contributions

HH made substantial contributions to the conception and design of the study, the acquisition of the data and the interpretation of the data. She drafted the final manuscript. JL and SR have been involved in drafting the manuscript and revising it critically for important intellectual content.

All authors have read and approved the final manuscript.

## References

[B1] Martimo K-P Audit matrix for evaluating Finnish occupational health units Scandinavian Journal of Work, Environment & Health 1998 24 439 443 9869317

[B2] Kimanen A Manninen P Räsänen K Rautio M Husman P Huskam K Factors associated with visits to occupational health physicians in Finland Occupational medicine 2010 60 1 29 35 10.1093/occmed/kqp128 19734240

[B3] Leino T Husman K Lamberg M 2006: Occupational Health services in Finland International supplement of Occupational Health Physician Journal 2006 2 22 23

[B4] Fletcher DJ Management of Multisite Occupational Health Services JOM 1994 36 4 434 437 8014715

[B5] Philipp R Goorman G Harling K Beattie B Study of business ethics in occupational medicine Occupational and Environmental Medicine 1997 54 351 356 10.1136/oem.54.5.351 9196458 PMC1128784

[B6] Premfors R 'Reshaping the democratic state: Swedish experiences in a comparative perspective' Public Administration 1998 77 141 159

[B7] Clark D Public Service Reform: A Comparative West European Perspective West European Politics 2002 23 25 44

[B8] Peters B "Policy Transfers Between Governments: The Case of Administrative Reforms" West European Politics 1997 20 71 88 10.1080/01402389708425218

[B9] Farnham D Horton S Barlow S Hondeghem S (eds) New Public Managers in Europe 1996 London: Macmillan

[B10] Flynn N Strehl F (eds) Public Sector Management in Europe 1996 London: Prentice Hall and Harvester Wheatsheaf

[B11] Kickert WJ Public Management and Administrative Reforms in Western Europe 1997 Cheltenham: Edward Elgar

[B12] Pollit C Summa H Trajectories of reform: Public Management change in four countries Public Money & Management 1997 17 7 1821883830

[B13] Pollit C Bouckaert G Public Management Reform: A Comparative Analysis Long Range Planning 2000 33 881 884 10.1016/S0024-6301(00)00083-2

[B14] Hood C Bekke H, Perry JL, Toonen TA Exploring Variations in Public Management Reforms of the 1990s Civil Service Systems in Comparative Perspective 1996 Bloomington: Indiana University Press

[B15] Esping-Andersen G The Three Worlds of Welfare Capitalism 1990 Cambridge: Polity Press

[B16] Huber E Stephens JD Partisan Governance, Women's Employment, and the Social Democratic Service State American Sociological Review 2000 65 323 342 10.2307/2657460

[B17] Green-Pedersen C New Public Management Reforms of the Danish and Swedish Welfare States: The Role of Different Social Democratic Responses. Governance An International Journal of Policy, Administration and Institutions 2002 15 271 294

[B18] Hoggett P "A New Public Management in the Public Sector?" Policy and Politics 1991 19 243 56 10.1332/030557391782454179

[B19] Hambleton R Hambleton R, Savitch RH, Murray S The New City Management Globalism and Local Democracy: Challenge and Change in Europe and North America 2002 New York: Palgrave

[B20] Ferlie E Ashburner L Pettigrew A The New Public Management in Action 1996 Oxford: Oxford University Press

[B21] Stark A 'What is the new public management' Journal of Public Administration Research and Theory 2002 12 1 137 151

[B22] Christensen M Yoshimi H Public Sector Performance Reporting: New Public Management and Contingency Theory Insights Government Auditing Review 2003 10 71 83

[B23] Lapsley I "Accounting and the new public management: instruments of substantive efficiency or a rationalising modernity?" Financial Accountability and Management 1999 15 201 207 10.1111/1468-0408.00081

[B24] Vakkuri J Meklin P Tulosmittaus ja "vastiketta rahalle" - ajattelutapa: Näkökulmia mittausteoreettisiin ongelmiin ja riskeihin Hallinnon tutkimus 1998 2 80 89

[B25] Pollit C Managerialism and the Public Services The Anglo-American Experience 1990 Oxford: Basil Blacwell Ltd, Oxford

[B26] Pollit C Beyond the Managerial Model: the Case for Broadening Performance Assesment in Government and Public Services Financial Accountability & Management 1986 2 3 155 170 10.1111/j.1468-0408.1986.tb00033.x21885098

[B27] van Wart M Berman E Contemporary Public Sector Productivity Values: narrower Scope, Tougher Standards and New Rules of the Game Public Productivity & Management Review 1999 22 3 326 347 10.2307/338070721893895

[B28] Boyne GA 'Processes, Performance and Best Value In Local Government' Local Government Studies 1999 25 1 15

[B29] Sanderson I Beyond Performance Measurement. Assessing "value" In Local Government Local Government Studies 1998 24 1 25

[B30] Lane J-E Lane J-E Introduction: Public sector reform: Only deregulation, privatisation and marketization Public Sector Reform: Rationale, Trends and Problems 1997 London: Sage 1 16

[B31] Aucoin P The New Public Management 1995 Canada; IRPP

[B32] Keating M Considine M, Painter M Managing for Results in the Public Interest Managerialism: The Great Debate 1997 Victoria: Melbourne University Press 112 133

[B33] Hartley J Innovation in Governance and Public Services: Past and Present Public Money & Management 2005 20 27 3421883830

[B34] Moore GC Henegan PM Defining and Prioritizing Public Performance Requirements. Public Performance Requirements Public Productivity & Management Review 1996 20 2 158 173 10.2307/338048321893895

[B35] DeLeon L Accountability in a "Reinvented Government" Public Administration 1998 76 539 558 10.1111/1467-9299.00116

[B36] Hood C A Public Management for all seasons Public Admin 1991 69 3 19 10.1111/j.1467-9299.1991.tb00779.x

[B37] Walsh K Public Services and Market Mechanisms Competition, Contracting, and the New Public Management 1995 Basingtoke and London

[B38] Lane J-E New public management 2001 London: Routledge

[B39] Pollit C The essential public manager 2003 Buckingham: Open University Press

[B40] Salminen A Julkisen toiminnan johtaminen: Hallintotieteen perusteet 2004 Helsinki: Edita

[B41] Vedung E Arviointiaalto ja sen liikkeelle panevat voimat 2003 Helsinki: STAKES, FinSoc työpapereita

[B42] Kolthoff E Huberts L van Den Heuvel H The Ethics of New Public Management: Is Integrity at Stake? PAQ 2006 30 399 439

[B43] Weikart LA The Giuliani Administration and the New Public Management in New York City Urban Affairs Review 2001 36 359 381 10.1177/10780870122184894

[B44] Moon MJ deLeon P Municipal Reinvention: Managerial Values and Diffusion among Municipalities Journal of Public Administration Research and Theory 2001 11 327 351

[B45] Box RC Running Government Like a Business Implications for Public Administration Theory and Practice The American Review of Public Administration 1999 29 19 43 10.1177/02750749922064256

[B46] Morris MH Jones FF Entrepreneurship in established organizations: The case of the public sector. Entrepreneurship Theory and Practice 1999 24 1 71 91

[B47] Exworthy M Halford S (eds) Professionalism and the New Managerialism in the Public Sector 1999 Buckingham: Open University Press

[B48] Ahonen P Salminen A Nordeuropaische Beitrage aus den Human- und Gesellschaftswissensschaften [Metamorphosis of the administrative welfare state: From depoliticization to political rationality] 1997 Frankfurt: Peter Lang GmbH

[B49] Piotrowski SJ Rosenbloom DH Nonmission-based Values in Results-oriented Public Management: The Case of Freedon of Information Public Administration Review 2002 62 643 657 10.1111/1540-6210.00247

[B50] Ezzamel M Hyndman N Johnsen Å Pallot J Has Devolution Increased Democratic Accountability? Public Money & Management 2004 24 145 152 10.1111/j.1467-9302.2004.00411.x21883830

[B51] Foster CP Plowden FJ The State Under Stress: Can the Hollow State be Good Government? 1997 Buckingham: Open University Press

[B52] Hays SF Kearney RC Riding a Crest of Wave: The National performance review and Public Management Reform International Journal of Public Administration 1997 20 11 40 10.1080/01900699708525187

[B53] Hood C "The New Public Management in the 1980's: variations on a theme" Accounting, Organizations and Society 1995 20 93 109 10.1016/0361-3682(93)E0001-W

[B54] Mascarenhas RC Building an enterprise culture in the public sector: Reforms in Australia, New Zealand and Great Britain Public Administration Review 1993 53 4 319 328 10.2307/977144

[B55] Pollit C Managerialism and the Public Services 1993 2 Oxford: Blackwell

[B56] Kettl D The global public management revolution 2005 Washington D.C: Brookings Institution Press

[B57] Manninen P (eds) Työterveyshuolto Suomessa 2007 2009 Helsinki: Työterveyslaitos

[B58] Terveydenhuollon menot ja rahoitus. Suomen virallinen tilasto, Terveys 2010 Terveyden ja hyvinvoinnin laitos 2010

[B59] Yksityiset terveyspalvelut 2007. Suomen virallinen tilasto, Terveys 2010 Terveyden ja hyvinvoinnin laitos 2010

[B60] Sosiaali- ja terveyspalvelujen henkilöstö 2007. Suomen virallinen tilasto, Terveys 2010 Terveyden ja hyvinvoinnin laitos 2010

[B61] Vuorenkoski L Mladovsky P Mossialos E Finland Health system review Health Systems in Transition 2008 10 4 1 168 31596240

[B62] Polit DF Hungler BP Nursing research Principles and Methods 1987 London: J.B. Lippincott Company

[B63] Fontana F Denzin NK, Lincoln YS The Interview. From Structured Question to Negotiated Text Handbook of Qualitative Research 2000 Thousand Oaks: Sage Publications 645 672

[B64] Marton F Phenomenography - describing conceptions of the world around us Instructional Science 1981 10 177 200 10.1007/BF00132516

[B65] Marton F Booth S Learning and Awareness 1997 New Jersey: Lawerence Erlbaum Associates

[B66] Lincoln YS Cuba EG Naturalistic Inquire 1985 Beverly Hills: Sage Publications

